# Functional Analysis of the Pepper Ethylene-Responsive Transcription Factor, CaAIEF1, in Enhanced ABA Sensitivity and Drought Tolerance

**DOI:** 10.3389/fpls.2017.01407

**Published:** 2017-08-22

**Authors:** Eunji Hong, Chae Woo Lim, Sang-Wook Han, Sung Chul Lee

**Affiliations:** ^1^Department of Life Science (BK21 Program), Chung-Ang University Seoul, South Korea; ^2^Department of Integrative Plant Science, Chung-Ang University Anseong, South Korea

**Keywords:** abscisic acid, CaAIEF1, drought stress, pepper, virus-induced gene silencing

## Abstract

Abscisic acid (ABA) is a plant hormone that plays a critical role in the response to environmental stress conditions, especially regulation of the stomatal aperture under water-deficit conditions. The signal transduction occurring during the stress response is initiated by transcription of defense-related genes. Here, we isolated the pepper ethylene-responsive transcription factor CaAIEF1 (*Capsicum annuum* ABA Induced ERF 1). The *CaAIEF1* gene was significantly induced after exposure to ABA, drought, and high salinity. Fusion of the acidic domain in the C-terminal region of CaAIEF1 to the GAL4 DNA-binding domain had a transactivation effect on the reporter gene in yeast. Further, the CaAIEF1-GFP fusion constructs localized in the nucleus. We used *CaAIEF1*-silenced plants and *CaAIEF1*-overexpressing (OX) plants to elucidate the biological function of CaAIEF1 in response to ABA and drought stress. *CaAIEF1*-silenced pepper plants and *CaAIEF1*-OX Arabidopsis plants displayed drought-sensitive and -tolerant phenotypes, respectively, which were characterized by regulation of transpirational water loss and stomatal aperture. In drought stress condition, quantitative RT-PCR analyses revealed that the expression levels of pepper stress-related genes were higher in *CaAIEF1*-silenced pepper plants than control plants. Moreover, expression levels of Arabidopsis stress-related genes were significantly reduced in *CaAIEF1*-OX plants compared with control plants in drought stress condition. Our findings suggest that CaAIEF1 positively regulates the drought stress response and the ABA signaling.

## Introduction

Plants are sessile organisms, and therefore, their growth and development is affected by abiotic stresses, including drought, high salinity, and extreme temperatures. Drought is a key abiotic stress that leads to loss of crop yield. Drought stress limits photosynthesis, reduces growth, and influences hormonal balance (Zhu, 2002). To adapt to drought stress conditions, plants have evolved various defense strategies to enhance water retention in cells via minimizing transpiration from the stomata and maximizing water uptake from the roots (Apse and Blumwald, 2002; Yamaguchi-Shinozaki and Shinozaki, 2006). The physiological mechanisms functioning under drought stress conditions have been intensively examined; however, the plant defense response is a complex phenomenon, and the precise adaptive mechanisms induced by drought stress remain elusive (Lee and Luan, 2012; Murata et al., 2015).

The major phytohormone abscisic acid (ABA) regulates many plant growth and development processes and abiotic stress responses (Cutler et al., 2010). Under drought stress conditions, ABA is biosynthesized in various tissues and accumulated in the leaf tissue, and this leads to induction of stress-adaptive mechanisms—including induction of defense-related genes, promotion of stomatal closure, and accumulation of various protective metabolites (Tan et al., 2003; Lee and Luan, 2012). A number of studies have indicated that the plant defense response to drought stress involves various mechanisms—from transcription to post-translational modification—and these mechanisms are influenced by the ABA signal transduction pathway (Lee and Luan, 2012).

Under stress conditions, defense-related genes are induced by the interaction between specific *cis*-acting elements and transcription factors (Thatcher et al., 2012; Llorca et al., 2014; Muller and Munne-Bosch, 2015). Transcription factors control the majority of defense-related genes and have several characteristics—such as transcriptional activation or repression and nuclear localization—that are essential for inducing or preventing target gene expression (Eulgem et al., 2000; Jakoby et al., 2002; Licausi et al., 2013; Llorca et al., 2014). In Arabidopsis, more than 1,800 transcription factors have been identified, and many of these transcription factors are involved in adaptation to stress conditions, including pathogens, drought and high salinity (Gong et al., 2004; Lee et al., 2006; Froidure et al., 2010; Mizoi et al., 2012; Fu and Dong, 2013). In particular, members of the APETALA2/Ethylene-Responsive Factor (AP2/ERF) transcription factor family are conservatively widespread in plants and are classified into five subfamilies according to the similarity and number of DNA-binding domains (Sakuma et al., 2002; Licausi et al., 2013). Previous studies have demonstrated that AP2/ERF transcription factors function as positive or negative regulators in the response to abiotic stress, and this facilitates plant adaptation to stress conditions. Thus, *AP2/ERF*-overexpressing (OX) plants exhibit tolerant or sensitive phenotypes after exposure to different stresses. For example, *ERF1*-OX Arabidopsis plants showed enhanced tolerance to drought and high salinity; these plants had increased levels of ABA and proline, which contribute to stress tolerance (Cheng et al., 2013). Activation of *AtERF7* inhibited ABA-responsive genes; hence, *AtERF7*-OX plants exhibited an ABA-hyposensitive and drought-sensitive phenotype (Song et al., 2005). The involvement of the AP2/ERF transcription factors in the plant adaptive response to abiotic stress is well established (Xu et al., 2011; Licausi et al., 2013; Muller and Munne-Bosch, 2015).

In this study, we characterized the drought-tolerant ERF transcription factor CaAIEF1 from *Capsicum annuum*. We used GAL4 fusion proteins in yeast to investigate the *in vitro* function of CaAIEF1 as a transacting factor. We evaluated the *in vivo* function of CaAIEF1 by examining the expression profiles of *CaAIEF1* in *CaAIEF1*-silenced pepper plants and *CaAIEF1*-overexpressing (OX) Arabidopsis plants in response to abiotic stresses. *CaAIEF1*-silenced pepper plants exhibited a drought-sensitive phenotype characterized by decreased stomatal closure and increased water loss. In contrast, *CaAIEF1*-OX plants displayed an drought-tolerant and ABA-hypersensitive phenotypes characterized by increased stomatal closure and low levels of transpirational water loss. Our findings imply that the CaAIEF1 transcription factor functions as a positive regulator of the defense response to drought stress.

## Materials and Methods

### Plant Materials

Seeds of pepper (*C. annuum*), *Arabidopsis thaliana* (ecotype Col-0), and tobacco (*Nicotiana benthamiana*) were sown in a soil mix, including sand, loam soil, vermiculite, perlite, peat moss (10:10:5:3:2, v:v:v:v:v). These plants were grown in a fixed condition at 24 ± 1°C (16 h/8 h, light/dark).

### Transactivation Analysis of CaAIEF1

The coding region of the *CaAIEF1* gene was cloned into the pGBKT7 vector, which includes a nuclear localization and GAL4 DNA-binding domains. We used *Saccharomyces cerevisiae* strain AH109 for CaAIEF1 transactivation analysis, which have two reporter genes (*Ade1* and *His3*) with *GAL4* promoters. The AH109 cells were transformed with pGBKT7 vector that carried *GAL4-CaAIEF1* fusion genes. The transformed yeast cells were selected into SD-adenine-histidine-leucine-tryptophan medium to confirm transcriptional activation.

### Virus-Induced Gene Silencing

For the loss-of function analysis of CaAIEF1, virus-induced gene silencing (VIGS) was conducted in pepper plants, as described by Lim et al. (2015b). Briefly, pTRV1 and pTRV2:*CaAIEF1* or pTRV2:00 transformed to *Agrobacterium tumefaciens* strain GV3101. The Agrobacterium cells were infiltrated by syringe to the pepper cotyledons (OD_600_ = 0.2 for each construct). Plants were grown in pepper growth condition described previously for spreading the virus.

### Generation of *CaAIEF1*-Overexpressing Arabidopsis Plants

For the elucidation of biological function of CaAIEF1, *CaAIEF1* overexpressing (OX) Arabidopsis was generated. The full-length coding region of *CaAIEF1* (accession no. KY652734) was inserted into pENTR/D-TOPO vectors (Invitrogen, Carlsbad, CA, United States). Through the LR reaction, the inserted genes were introduced into pK2GW7 to constitutively express each gene with the cauliflower mosaic virus (CaMV) 35S promoter (Karimi et al., 2002); the generated construct was transformed into *Agrobacterium tumefaciens* strain GV3101. The floral dip method was applied for transformation of Arabidopsis with the *CaAIEF1* gene (Clough and Bent, 1998). Overexpressing plants were selected by germinating putative transformed seeds on Murashige and Skoog (MS) plates supplemented with 50 μg⋅mL^-1^ of kanamycin as selection marker. Seeds of T3 plants were harvested from second-generation transgenic plants showing a 3:1 segregation ratio on MS plates supplemented with the same antibiotic.

### ABA, Dehydration, and NaCl Treatments

To analyze the expression patterns of *CaAIEF1* in pepper plants, which had been treated with ABA, NaCl, and dehydration, leaf samples were prepared as described by Lim et al. (2015b). For the ABA and NaCl treatment, the six-leaf-stage pepper plants were treated with 100 μM ABA, irrigated with a salt solution (200 mM). Whole plants were dried on 3-mm paper (Whatman, Clifton, United Kingdom), or the roots were removed and the aerial parts of plants were dried. After treatment, the third and fourth leaves were harvested at the several time points.

For the dehydration phenotype analysis, 5-week-old pepper plants and 3-week-old Arabidopsis plants were randomly placed and subjected to dehydration treatment by withholding watering for 12 and 11 days, respectively. After re-watering, the survival rate was calculated by counting the plant number with resumed growth. To determine the dehydration tolerance in a quantitative manner, rates of water loss were measured by drying leaves detached from gene-silenced pepper plants and Arabidopsis transgenic plants.

For quantitative real-time transcription-polymerase chain reaction (qPCR) analysis, 4-week-old wild-type and *CaAIEF1*-OX Arabidopsis were treated with dehydration stress, and harvested at the several time points.

### Measurement of Stomatal Aperture and Leaf Temperature

The measurements of stomatal pore size and leaf temperature were performed as described previously (Lim and Lee, 2016). Briefly, pepper and Arabidopsis leaf peels were floated in stomatal opening buffer (SOS: 50 mM KCl and 10 mM MES-KOH, pH 6.15, 10 mM CaCl_2_) in the light condition for 2.5 h. Stomatal closure was induced by replacing the buffer with fresh SOS containing various concentrations of ABA. After an additional 2.5 h of incubation in each buffer, 100 stomata per each sample were observed under a Nikon Eclipse 80i microscope. An infrared camera (FLIR systems; T420) were used for obtaining the thermal images, and FLIR Tools+ ver 5.2 software measured the leaf temperatures.

### Quantitative Real-time Transcription-Polymerase Chain Reaction

RNA samples were digested with RNA-free DNase to inhibit contamination of genomic DNA. The cDNA were synthesized using a Transcript First Strand cDNA Synthesis kit (Roche, Indianapolis, IN, United States). For qPCR analysis, the specific primers and CFX96 Touch^TM^ Real-Time PCR detection system (Bio-Rad, Hercules, CA, United States) were used (Supplementary Table S1). The PCR was programmed as follows: 95°C for 5 min; 45 cycles each at 95°C for 20 s and 60°C for 20 s; and 72°C for 20 s. To determine relative expression level, we used the ΔΔCt method (Livak and Schmittgen, 2001). For the normalization, the Arabidopsis *AtACT8* and pepper *CaACT1* genes were used.

### Subcellular Localization of CaAIEF1

CaAIEF1-GFP proteins were expressed in the leaves of *N. benthamiana* epidermis cells by using infiltration of *Agrobacterium tumefaciens* strain GV3101 with strain carrying p19 suppressor (1:1 ratio; OD_600_ = 0.5). The GFP signal was observed 2 days after infiltration, using a confocal microscope (510 UV/Vis Meta; Zeiss, Oberkochen, Germany).

## Results

### Isolation of Ethylene-Responsive Transcription Factor CaAIEF1

**FIGURE 1:**
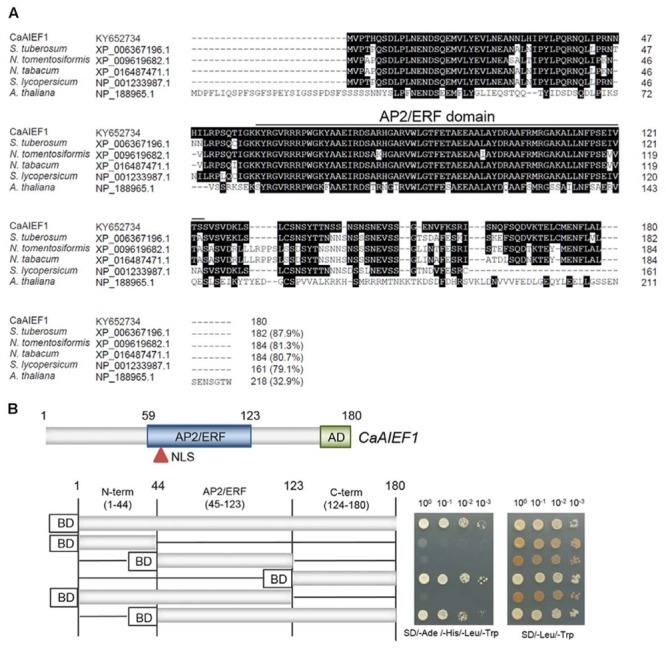
Molecular characterization of the CaAIEF1 (*Capsicum annuum* ABA Induced ERF 1) protein. **(A)** Comparisons of the deduced amino acid sequence of the CaAIEF1 protein with those of the *Solanum tuberosum* (accession no. XP_006367196.1), *Nicotiana tomentosiformis* (accession no. XP_009619682.1), *N. tabacum* (accession no. XP_016487471.1), *Solanum lycopersicum* (accession no. XP_001233987.1) and *Arabidopsis thaliana* (accession no. NP_188965.1) proteins. Identical amino acid residues are highlighted in black, and the upper line indicates the AP2/ERF domain. **(B)** Transactivation of GAL4-responsive transcription by the full-length and truncated forms of the *CaAIFF1* gene using a GAL4 yeast system. Derivatives of *S. cerevisiae* strain AH109 harboring plasmids that encode GAL4-CaAIFF1 were grown on SD medium lacking adenine, histidine, leucine, and tryptophan (SD-adenine-histidine-leucine-tryptophan; left) or leucine and tryptophan (SD-leucine-tryptophan, right). Plates were incubated at 28°C for 5 days. pGBKT7 indicates the vector used in this experiment; this vector expressed GAL4 BD.

To identify the novel ABA-induced transcription factor, we conducted an RNA-seq assay using control and ABA treated pepper leaves; we identified the putative pepper ABA-induced ERF. We designated gene name *CaAIEF1* (*C. annuum* ABA Induced ERF 1) by domain analysis (**Figure 1A** and **Supplementary Figure S1**). *CaAIEF1* encoded 180 amino acids. The predicted protein consisted of an AP2/ERF domain with 65 amino acids (59–123) in the central region and an acidic domain (AD) consisting of 21 amino acids (160–180); these domains specifically bind to promoter regions and activate target genes (**Figure 1A**) (Licausi et al., 2013). Moreover, a putative nuclear localization signal (NLS) was detected in the AP2/ERF domain. Amino acid sequence alignments of CaAIEF1 with ERF proteins revealed that these ERF protein members have a highly conserved AP2/ERF domain. CaAIEF1 shares identity with other ERF proteins of *Solanum tuberosum* (accession no. XP_006367196.1, 87.9% identity), *N. tomentosiformis* (accession no. XP_009619682.1, 81.3% identity), *N. tabacum* (accession no. XP_016487471.1, 80.7% identity), *Solanum lycopersicum* (accession no. XP_001233987.1, 79.1% identity) and *A. thaliana* (accession no. NP_188965.1, 32.9% identity). However, CaAIEF1 does not share any identity with pepper ERF proteins (**Supplementary Figure S2**).

Transcription factors have an activation domain, which functions in activation of the target gene. The activation domain is enriched in glutamine, proline, or acidic amino acids (Schwechheimer et al., 1998a,b; Jakoby et al., 2002; Cantin et al., 2003). Transactivation of acidic domains has universal functions in eukaryotic transcription factors (Hahn, 1993; Schwechheimer et al., 1998a). CaAIEF1 contains an acidic domain in the C-terminal region; hence, we used deletion constructs of the *CaAIEF1* gene to determine whether the CaAIEF1 protein functions as a transactivation factor in yeast. The deletion constructs were inserted into the pGBKT7 vector carrying the GAL4 DNA-binding domain, expressed in *S. cerevisiae* strain AH109 (**Figure 1B**). Yeast cells carrying the C-terminal region (containing the acidic domain) grew well in the selection medium, suggesting activation of the reporter genes. Our results indicate that the C-terminal region of CaAIEF1 act as a potential transcriptional activator.

### Expression of the *CaAIEF1* Gene and Subcellular Localization of the CaAIEF1 Protein

**FIGURE 2:**
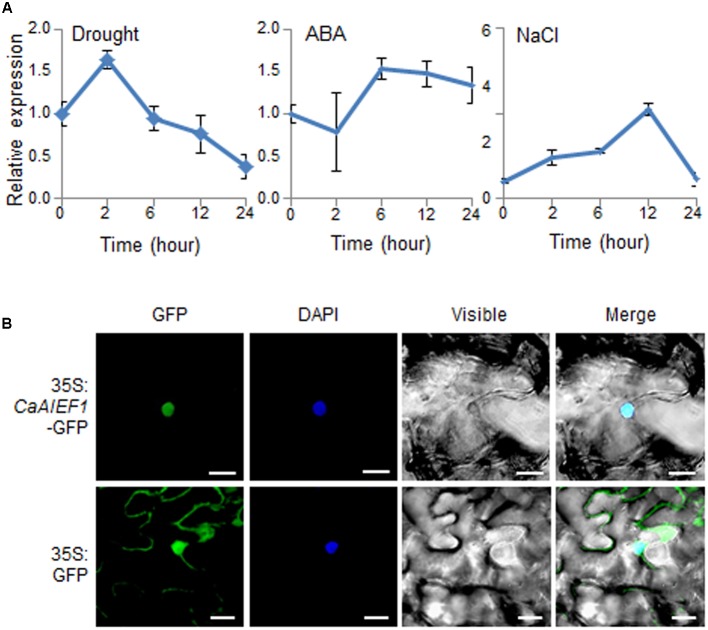
Expression of *CaAIEF1* and subcellular localization of CaAIEF1. **(A)** The expression pattern of the *CaAIEF1* gene was analyzed in the leaves of pepper plants after treatment with 100 μM abscisic acid (ABA), drought, or 200 mM NaCl. The pepper *Actin1* gene was used as an internal control. **(B)** Subcellular localization of the 35S: *CaAIEF1*-GFP fusion protein in *N. benthamiana* epidermal cells. The 35S: *CaAIEF1*-GFP construct was expressed via agroinfiltration of *N. benthamiana* leaves and observed using a confocal laser-scanning microscope. 4′,6-Diamidino-2-phenylindole (DAPI) staining was used as a marker for the nucleus. The scale bar represents 20 μm.

To examine whether *CaAIEF1* is expressed in response to several stresses, including drought, ABA, and NaCl, we performed quantitative RT-PCR analysis (**Figure 2A**). In drought-treated pepper leaves, the expression level of *CaAIEF1* was up-regulated at 2 h and thereafter gradually decreased. ABA is a key abiotic stress-related hormone involved in stress signal transduction—especially under drought stress conditions (Lee and Luan, 2012). In ABA-treated pepper leaves, *CaAIEF1* was induced within 6–24 h. *CaAIEF1* transcripts started to induce at 2 h and reached peak levels after 12 h by NaCl treatment. The CaAIEF1 is ethylene responsive factor; hence, we checked whether *CaAIEF1* is induced by ethylene. As shown in **Supplementary Figure S3A**, the transcription level of *CaAIEF1* was up-regulated by ethylene. Moreover, the *CaAIEF1* expression was also regulated by developmental stages (**Supplementary Figure S3B**).

CaAIEF1 has an NLS in the AP2/ERF domain of the central region (amino acids 202–219); hence, we predicted that CaAIEF1 localizes in the nucleus. To confirm the subcellular localization of the CaAIEF1 protein (**Figure 2B**), we used the *CaAIEF1* coding region with green fluorescent protein (GFP) cDNA. GFP fluorescence signals were detected in the nucleus of *N. benthamiana* cells, indicating that CaAIEF1 functions in the nucleus.

### Decreased Tolerance of *CaAIEF1*-Silenced Pepper Plants in Response to Drought Stress

**FIGURE 3:**
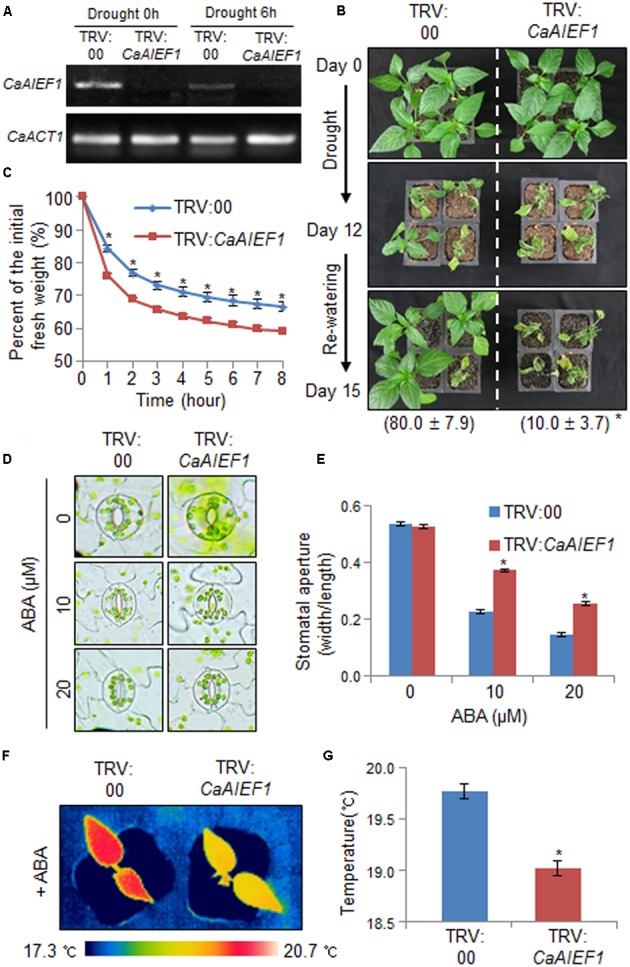
Reduced tolerance of *CaAIEF1*-silenced pepper plants to drought stress. **(A)** Reverse transcription-polymerase chain reaction (RT-PCR) analysis of *CaAIEF1* expression in *CaAIEF1*-silenced pepper plants in normal and drought stress conditions. The pepper Actin1 (*CaACT1*) gene was used as an internal control. **(B)** Drought sensitivity of control (TRV2:00) and *CaAIEF1*-silenced pepper (TRV2:*CaAIEF1*) plants. Four-week-old plants of each line were subjected to drought stress by withholding watering for 12 days. The plants were re-watered for 3 days, after which representative images were taken and the percentages of surviving plants were calculated. Data represent the mean ± standard error of three independent experiments, each evaluating 30 plants. **(C)** Transpirational water loss from the leaves of control and *CaAIEF1*-silenced pepper plants. Leaves were detached and the fresh weights of each line were measured every hour. Data represent the mean ± standard error of three independent experiments, each evaluating 30 plants. **(D,E)** ABA-induced stomatal closure in control and *CaAIEF1*-silenced pepper plants. Leaf peels were incubated with 10 and 20 μM ABA, after which representative images were taken **(D)**, and the stomatal apertures of each line were measured **(E)**. **(F,G)** Leaf temperatures of control and *CaAIEF1*-silenced pepper plants after exposure to ABA. Control and *CaAIEF1*-silenced pepper plants were sprayed with 50 μM ABA. After 6 h, representative thermographic images were taken **(F)**, and the mean leaf temperature was measured using the first and second leaves of each line (*n* = 18) **(G)**. All data represent the mean ± SE of three independent experiments. Asterisks indicate significant differences (Student’s *t*-test; *P* < 0.05).

The expression level of *CaAIEF1* was induced by ABA and abiotic stresses; hence, we investigated the *in vivo* function of CaAIEF1 using virus-induced gene silencing (VIGS) in pepper plants and Arabidopsis transgenic plants. Semi-quantitative RT-PCR analysis revealed that *CaAIEF1* transcripts were not expressed in *CaAIEF1*-silenced pepper plants in normal and drought stress conditions (**Figure 3A**). We investigated the biological function of CaAIEF1 in drought stress condition (**Figure 3B**). Under well-grown conditions, we did not observe any phenotypic differences in both plants (**Figure 3B**, upper panel). However, when we subjected pepper plants to dehydration for 12 days and rehydration for 3 days, *CaAIEF1*-silenced pepper plants showed a more wilted phenotype than control plants (**Figure 3B**, middle and lower panels). In addition, after rehydration, the 80% of control and only 10.0% of *CaAIEF1*-silenced pepper plants resumed their growth. To examine whether the drought-sensitive phenotype was cause by rapid transpirational water loss, the fresh weight of rosette leaves were measured during 8 h after detachment (**Figure 3C**). The higher transpirational water loss was detected in *CaAIEF1*-silenced plants than in control plants. Under dehydrated conditions, ABA is synthesized in several tissues and accumulates in the leaves; this leads to stomatal closure via regulation of guard cell turgor pressure (Lee and Luan, 2012). To examine the biological role of CaAIEF1 in drought stress-induced ABA signaling, we measured the stomatal apertures and leaf temperatures of control and *CaAIEF1*-silenced pepper plants with or without ABA (**Figures 3D–G**). In the absence of ABA, we did not observe differences between control and *CaAIEF1*-silenced pepper plants. However, after exposure to 10 and 20 μM ABA, the stomatal pore sizes were large in *CaAIEF1*-silenced pepper plants compared with control plants (**Figures 3D,E**). Moreover, the leaf temperatures were significantly lower in *CaAIEF1*-silenced plants than in control pepper plants, implying that increased evaporative cooling was caused by decreased stomatal closure (**Figures 3F,G**). Our data indicated that the decreased ABA sensitivity of *CaAIEF1*-silenced pepper plants leads to increased water loss and a drought-sensitive phenotype.

### Hypersensitivity of *CaAIEF1*-OX Plants in Response to ABA

**FIGURE 4:**
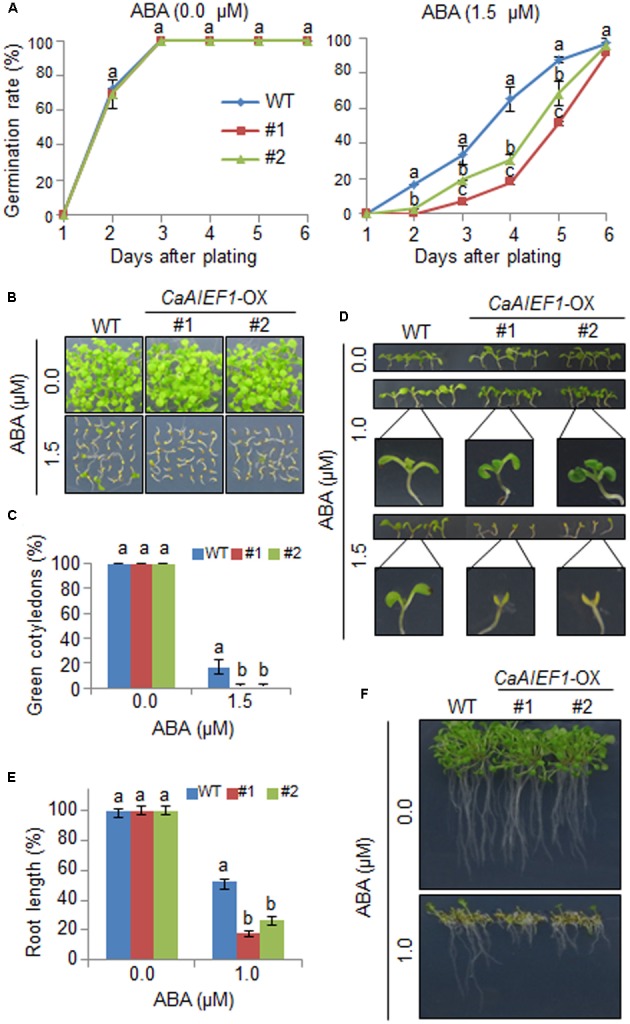
Increased sensitivity of *CaAIEF1*-overexpressing (OX) transgenic Arabidopsis plants to ABA during germination and seedling growth. **(A)** Seed germination of wild-type (WT) and transgenic lines in response to ABA. Seeds were germinated on 0.5× Murashige and Skoog (MS) agar plates containing 0.0 μM or 1.5 μM ABA. Data represent the mean ± standard error values obtained after evaluating 150 seeds from three independent experiments. **(B–D)** Growth of WT and transgenic seedlings on 0.5× MS agar plates containing various concentrations of ABA. Representative photographs were taken 12 days after plating **(B,C)**. Quantification of green cotyledons in the wild-type and each mutant line was performed 12 days after plating **(D)**. Data represent the mean ± standard error values obtained after evaluating 72 seeds from three independent experiments. **(E,F)** Root elongation of WT and transgenic plants in response to ABA. The root length of each plant was measured 9 days after plating. Data represent the mean ± standard error of three independent experiments. Different letters indicate significant differences (ANOVA; *P* < 0.05).

To further examine the biological function of CaAIEF1, we generated *CaAIEF1*-OX Arabidopsis plants. Semi-quantitative RT-PCR analysis revealed that *CaAIEF1* was only expressed in *CaAIEF1*-OX plants (**Supplementary Figure S4**). CaAIEF1 is induced by ABA; hence, we predicted that enhanced expression of *CaAIEF1* modulates ABA signaling. To examine whether CaAIEF1 influences the ABA-mediated signaling, we analyzed the ABA sensitivity during the germinative- and post-germinative stages, including seed germination, seedling establishment, and root growth (**Figure 4**). We did not observe marked differences in germination rates between both plants on growth media without ABA. In the presence of 1.5 μM ABA, *CaAIEF1*-OX seeds germinated slower than wild-type seeds (**Figure 4A**). Moreover, *CaAIEF1*-OX plants were hypersensitive to ABA at seedling stages in seedling establishment (**Figures 4B–D**) root growth (**Figures 4E,F**). To determine whether ABA hypersensitivity in root growth and seedling establishment in *CaAIEF1*-OX plants is caused by late germination, the seedlings at 3 days after germination in the absence of ABA were transferred to medium including ABA. As shown in **Supplementary Figure S5**, the *CaAIEF1*-OX plants also exhibited ABA sensitive phenotype. The ABA-hypersensitive phenotype displayed by *CaAIEF1*-OX plants indicates that CaAIEF1 modulates the ABA-mediated response.

### Increased Tolerance of *CaAIEF1*-OX Plants in Response to Drought Stress

**FIGURE 5:**
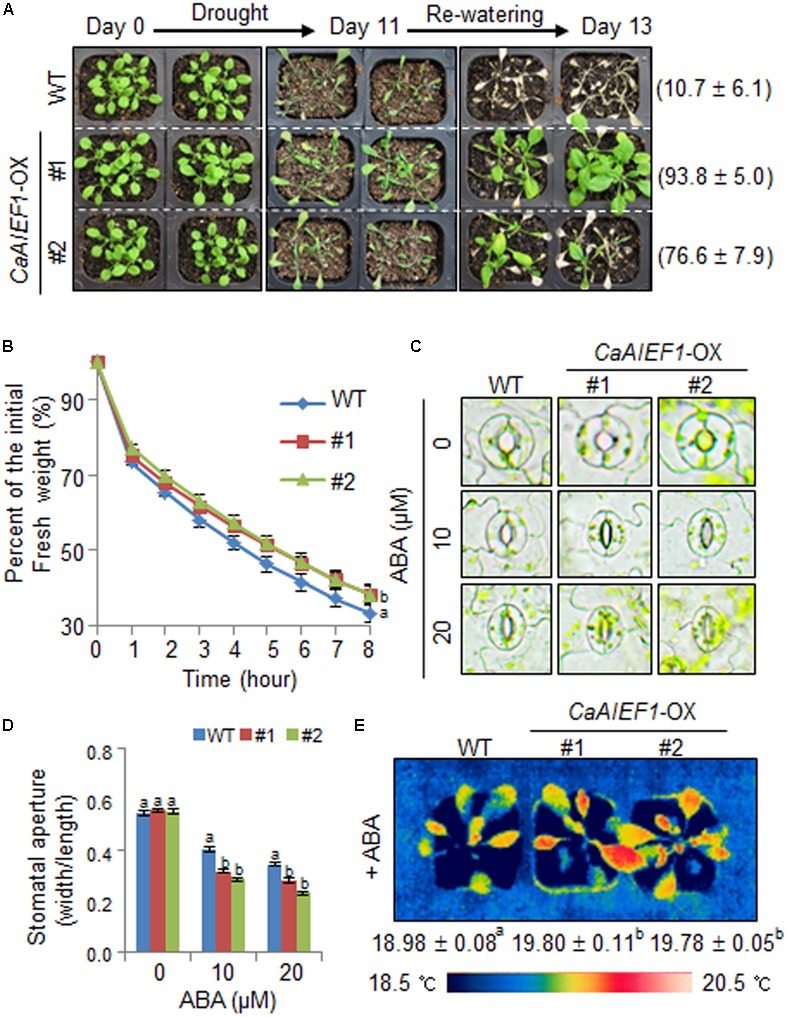
Increased tolerance of *CaAIEF1*-OX plants to drought stress. **(A)** Drought-tolerant phenotype of *CaAIEF1*-OX transgenic plants. Four-week-old wild-type (WT) and transgenic plants were subjected to drought stress by withholding watering for 11 days and then re-watering for 2 days. Representative images were taken before (left) and after (middle) drought and after 2 days of re-watering (right). Survival rates of plants were calculated after 2 days of re-watering. Data represent the mean ± standard error of three independent experiments, each evaluating 20 plants. **(B)** Transpirational water loss from the leaves of WT and transgenic plants at various time points after detachment of rosette leaves. Data represent the mean ± standard error of three independent experiments, each evaluating 50 leaves. **(C,D)** Stomatal apertures in WT and *CaAIEF1*-OX plants treated with ABA. Leaf peels were harvested from 4-week-old plants of each line and incubated in stomatal opening solution (SOS) buffer containing the indicated concentrations of ABA. Representative images were taken under a microscope **(C)** and the stomatal apertures were measured **(D)**. Data represent the mean ± standard error of three independent experiments. **(E)** Increased leaf temperatures of *CaAIEF1*-OX plants in response to ABA treatment. Data represent the mean ± standard error of three independent experiments, each evaluating 10 plants. Different letters indicate significant differences (ANOVA; *P* < 0.05).

*CaAIEF1*-silenced pepper plants showed a drought-sensitive phenotype; hence, we examined whether *CaAIEF1*-OX plants exhibit drought tolerant phenotype (**Figure 5**). Under well-grown conditions, we did not observe phenotypic differences in both plants (**Figure 5A**, left panel). However, when we treated drought stress by dehydration for 11 days and rehydration for 2 days, *CaAIEF1*-OX plants showed a less wilted phenotype than wild-type plants (**Figure 5A**, middle and right panels). In addition, the survival rate of *CaAIEF1*-OX plants (76.6–93.8%) was higher than that of wild-type plants (10.7%). To examine whether different transpirational water loss leads to altered drought tolerance, the leaf fresh weight was measured (**Figure 5B**). In accordance with the drought tolerance, the transpirational water loss was lower in *CaAIEF1*-OX plants than in wild-type plants. To examine the biological role of CaAIEF1 in drought stress-induced ABA signaling, we measured the stomatal apertures and leaf temperatures of wild-type and *CaAIEF1*-OX plants with or without ABA (**Figures 5C–E**). We did not observe any differences between both plants. ABA treatment led to an increase in stomatal closure in wild-type and transgenic plants. However, after exposure to 10 and 20 μM ABA, the stomatal pore sizes were small in *CaAIEF1*-OX plants compared with wild-type plants (**Figures 5C,D**). We measured leaf temperatures, as indicator of stomatal opening/closure via evaporative cooling. The leaf temperatures were significantly higher in *CaAIEF1*-OX plants than in wild-type plants (**Figure 5E**). Our results imply that the ABA hypersensitivity displayed by *CaAIEF1*-OX plants leads to decreased water loss and contributes to a drought-tolerant phenotype.

**FIGURE 6:**
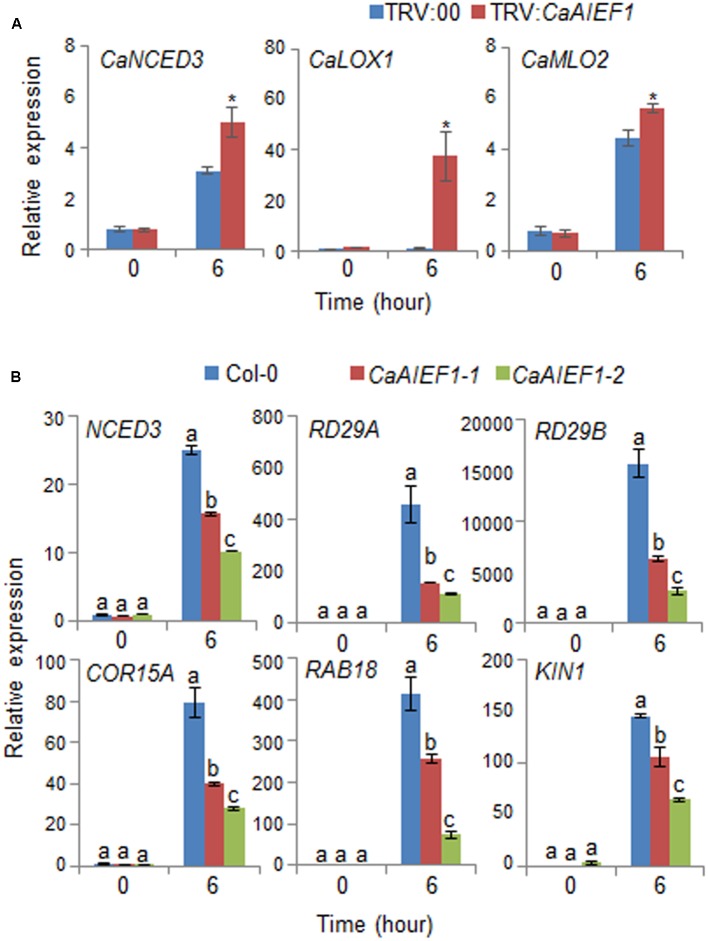
Quantitative real-time polymerase chain reaction (qPCR) analysis of stress-responsive genes in the leaves of *CaAIEF1*-silenced pepper plants **(A)** and *CaAIEF1*-OX transgenic Arabidopsis **(B)** plants exposed to drought stress; analysis was performed 6 h after detachment. The relative expression levels (ΔΔCT) of each gene were normalized to the geometric mean of pepper *Actin1* and Arabidopsis *Actin8* as internal control genes. Data represent the mean ± standard error of three independent experiments. Different letters indicate significant differences between wild-type and transgenic lines (*P* < 0.05; ANOVA followed by Fisher’s LSD test).

Several previous studies suggested that the expression of stress-responsive genes influence stress tolerance or stress sensitivity; hence, we examined whether stress-responsive genes in pepper and Arabidopsis are directly or indirectly regulated by the expression level of *CaAIEF1* (**Figure 6**). We subjected plants to dehydration stress by removing the roots from the soil. Contrary to our expectations, q-PCR assay revealed that the induction of stress-responsive genes was higher in *CaAIEF1*-silenced pepper leaves than in control pepper leaves (**Figure 6A**). However, the induction of several stress-responsive genes, including *NCED3*, *RD29A*, *RD29B*, *COR15A*, *RAB18*, and *KIN1*, was lower in *CaAIEF1*-OX leaves than in wild-type leaves (**Figure 6B**). Our data imply that *CaAIEF1* expression negatively affected the expression of stress-responsive genes.

## Discussion

The plant hormone ABA plays a critical role in adaptive response to environmental stresses. Plant response and adaptation to abiotic stress is dependent on the several hormone signals, to regulate the various transcription factor expressions that enable plants to adapt specific stress. The results of our present study suggest that CaAIEF1 is one of ABA signaling component in pepper plants. Many regulatory genes—including transcription factors, protein kinase, and phosphatase—have been identified as essential components of the molecular network in ABA signaling, and the involvement of transcription factors in the stress response is well established (Seo and Koshiba, 2002; Cutler et al., 2010; Wind et al., 2013; Ding et al., 2015; Tian et al., 2015; Li et al., 2016). Several previous studies have demonstrated that the target genes of transcription factors are activated or repressed and that expression or silencing of these genes is the cue for regulation of the stress response. For example, the ABI3—which is upstream of ABI5 in the ABA response—activates ABI5 transcription, leading to acquired osmotic stress tolerance (Lopez-Molina et al., 2002). Ethylene modulates plant growth and development as well as defense response to environmental stresses. In previous studies, several AP2/ERF transcription factors are involved in response to stress signaling (Licausi et al., 2013; Wang et al., 2015a,b). Transcription factors contain at least one DNA-binding domain, which specifically adheres to a distinct promoter region of target genes. In general, plant transcription factors have activation domains, which contain specific amino acids rich region, such as glutamine, proline, and acidic amino acids (Licausi et al., 2013). AP2-containing transcription factors function as transcription activators or repressors, which usually contain activation or repression domain, respectively (Ikeda and Ohme-Takagi, 2009; Licausi et al., 2013). The repression domain accords repression to the transcription factor by binding to the promoter of target genes (Ohta et al., 2001). CaAIEF1 contains an AP2 domain for DNA binding and an acidic domain for transactivation (**Figure 1**); however, we were unable to find a repression motif. Our results imply that CaAIEF1 functions as a transcription activator in yeast system.

The transcripts of ABA biosynthesis-, ABA signaling-, and defense responsive-genes are essential for plant adaptation and survival under dehydrated conditions (Zhang et al., 2006; Aubert et al., 2010; Hubbard et al., 2010; Fujita et al., 2011; Lim et al., 2015a). *CaAIEF1*-silenced pepper plants and *CaAIEF1*-OX transgenic Arabidopsis plants were used to elucidate the biological function of CaAIEF1. The transformation efficiency was very low in pepper plants; hence, a VIGS analysis in pepper plants and overexpression in Arabidopsis were used to investigate the biological functions of *CaAIEF1. CaAIEF1*-silenced pepper plants exhibited an ABA-insensitive and drought-sensitive phenotypes characterized by reduced stomatal closure and low leaf temperatures (**Figure 3**). *CaAIEF1*-OX Arabidopsis plants displayed a contrasting phenotype to that of *CaAIEF1*-silenced pepper plants (**Figures 4**, **5**). Under abiotic stress conditions, ABA biosynthesis-related genes—including *NCED3*—are induced, and ABA is produced in various plant tissues, leading to the defense response (Iuchi et al., 2001). Previous studies have shown that altered expression of defense responsive genes is closely associated with stress tolerance and sensitivity (Verslues and Bray, 2006; Shinozaki and Yamaguchi-Shinozaki, 2007). Therefore, we predicted that under dehydration, the expression levels of defense responsive genes in *CaAIEF1*-silenced pepper plants and *CaAIEF1*-OX Arabidopsis plants are down-regulated and up-regulated, respectively. However, contrary to our prediction, the transcription of defense responsive genes in *CaAIEF1*-silenced pepper and *CaAIEF1*-OX Arabidopsis plants were up-regulated and down-regulated, respectively. If *CaAIEF1*-silenced pepper plants do not have the mechanisms to initiate a successful defense response, these plants are not able to alleviate drought stress and therefore cannot adapt to these stress conditions; hence, the drought stress signal is continually transferred to various tissues, leading to defense responsive gene expression. Moreover, this stress signal also induces defense response-related gene expression. Urano et al. (2009) demonstrated that *NCED3* positively modulates the expression of defense responsive genes; therefore, up- or down-regulation of *NCED3* affects the transcription of defense responsive genes. Moreover, CaAIEF1 may regulate the transcription of different stress-related genes; however, the precise target genes of this transcription factor remain elusive.

## Conclusion

The CaAIEF1 protein localizes to the nucleus, where it presumably acts as a transcriptional regulator. Loss-of function of *CaAIEF1* in pepper plants led to increased drought sensitivity, whereas ectopic expression of *CaAIEF1* in Arabidopsis induced ABA hypersensitivity and drought tolerance. Taken together, our data indicate that CaAIEF1 positively regulates the defense response to drought stress. The precise mechanisms involved in the activation of the defense response by CaAIEF1 remain unclear, and further studies to identify the downstream target genes of this transcription factor are required.

## Author Contributions

EH and CWL performed experiments and analyzed the results. S-WH and SCL designed the experiments and wrote the manuscript.

## Conflict of Interest Statement

The authors declare that the research was conducted in the absence of any commercial or financial relationships that could be construed as a potential conflict of interest.

## Supplementary Material

FIGURE S1 Sequence logo drawn based on the alignment of amino acid sequences of AP2/ERF domain. The overall height of the stack indicates the sequence conservation at that position, while the height of symbols within the stack indicates the relative frequency of each amino at that position.

FIGURE S2 Comparisons of the deduced amino acid sequence of the CaAIEF1 protein with *Capsicum annuum* proteins. Identical amino acid residues are highlighted in black, and the upper line indicates the VIGS target region.

FIGURE S3 Expression of *CaAIEF1*. **(A)** The expression pattern of the *CaAIEF1* gene was analyzed in the leaves of pepper plants after treatment with ethylene (10 μL/L). **(B)** The expression pattern of the *CaAIEF1* gene was analyzed in the leaves of pepper plants in developmental stages. The pepper *Actin1* gene was used as an internal control.

FIGURE S4 Expression of the *CaAIEF1* gene in transgenic Arabidopsis plants. Reverse transcription-polymerase chain reaction (RT-PCR) analysis of *CaAIEF1* expression in transgenic Arabidopsis plants overexpressing *CaAIEF1*. The expression level of *CaAIEF1* was analyzed in the leaves of 4-week-old *CaAIEF1*-OX Arabidopsis plants. The Arabidopsis *Actin8* gene was used as an internal control.

FIGURE S5 Increased sensitivity of *CaAIEF1*-OX transgenic Arabidopsis plants to abscisic acid (ABA) during post-germinative stage. **(A)** Root elongation of wild type and transgenic plants exposed to ABA after germination. Three-day-old seedlings grown on 0.5X MS were transferred to 0.5X MS containing 0, 10, or 30 μM ABA. After 9 days, the representative images were taken. **(B)** Growth of wild-type and transgenic plants exposed to ABA after germination. Two-day-old seedlings grown on 0.5X MS were transferred to 0.5X MS containing 0 or 5 μM ABA. After 4 days, the representative images were taken, and the cotyledon greening in each line was measured. Data represent the mean ± standard error values obtained after evaluating 50 plants from three independent experiments. Different letters indicate significant differences (ANOVA; *P* < 0.05).
