# Supplementary figures and images for: Functional Analysis of the Pepper Ethylene-Responsive Transcription Factor, CaAIEF1, in Enhanced ABA Sensitivity and Drought Tolerance
Source: Front Plant Sci. 2017 Aug 22;8:1407. doi: 10.3389/fpls.2017.01407 (PMC5572256; doi:10.3389/fpls.2017.01407)

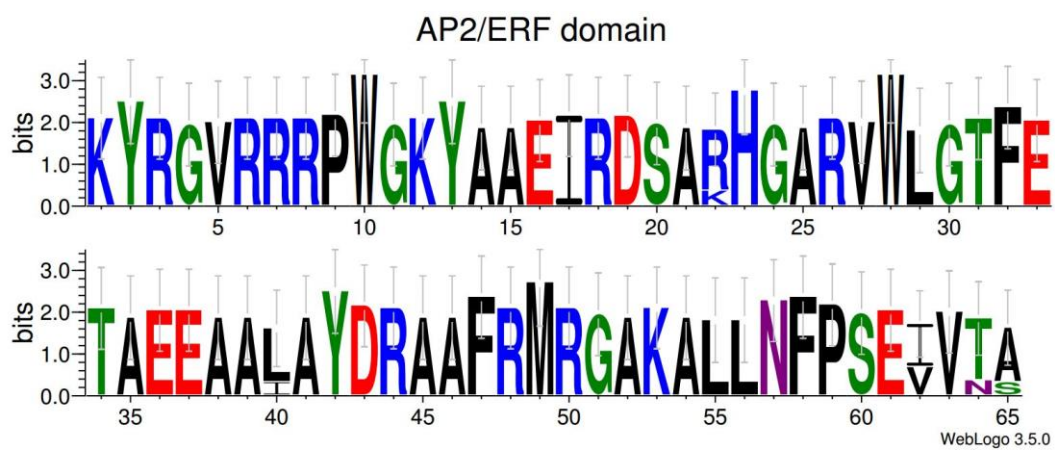

Supplementary Fig. S1. Hong et al.

Supplement: FIGURE S1 — Sequence logo drawn based on the alignment of amino acid sequences of AP2/ERF domain. The overall height of the stack indicates the sequence conservation at that position, while the height of symbols within the stack indicates the relative frequency of each amino at that position. [file Image_1.PDF]

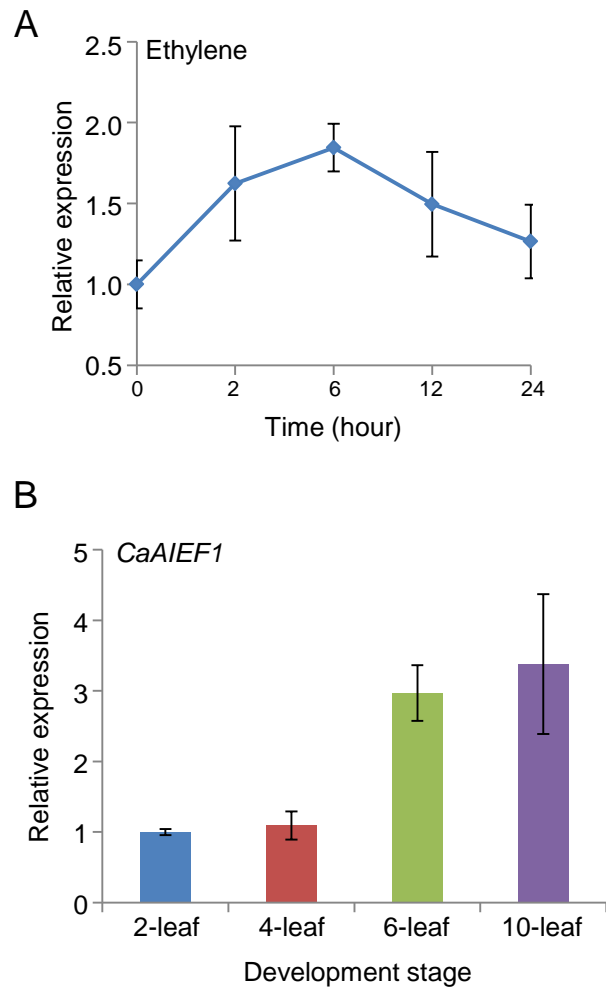

Supplementary Fig. S3. Hong et al.

Supplement: FIGURE S3 — Expression of CaAIEF1. (A) The expression pattern of the CaAIEF1 gene was analyzed in the leaves of pepper plants after treatment with ethylene (10 μL/L). (B) The expression pattern of the CaAIEF1 gene was analyzed in the leaves of pepper plants in developmental stages. The pepper Actin1 gene was used as an internal control. [file Image_3.PDF]

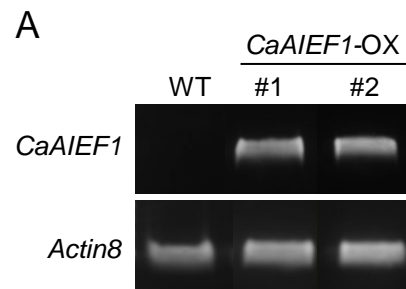

Supplementary Fig. S4. Hong et al.

Supplement: FIGURE S4 — Expression of the CaAIEF1 gene in transgenic Arabidopsis plants. Reverse transcription-polymerase chain reaction (RT-PCR) analysis of CaAIEF1 expression in transgenic Arabidopsis plants overexpressing CaAIEF1. The expression level of CaAIEF1 was analyzed in the leaves of 4-week-old CaAIEF1-OX Arabidopsis plants. The Arabidopsis Actin8 gene was used as an internal control. [file Image_4.PDF]

A

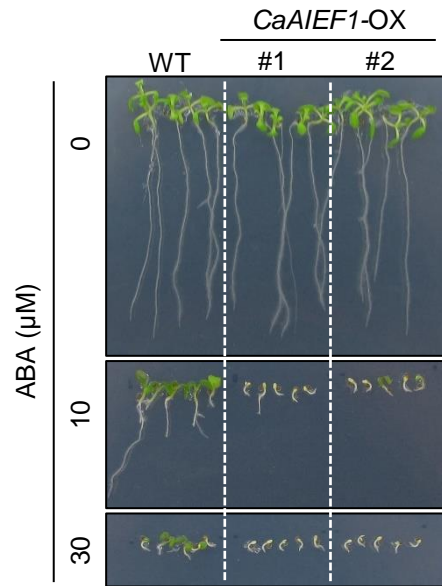

B

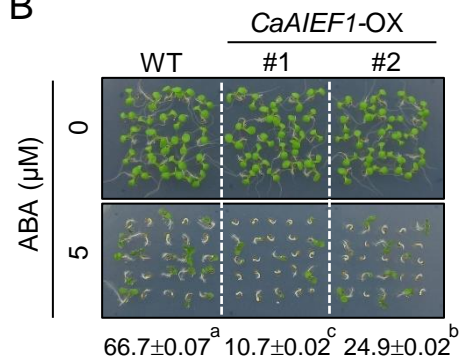

Supplementary Fig. S5. Hong et al.

Supplement: FIGURE S5 — Increased sensitivity of CaAIEF1-OX transgenic Arabidopsis plants to abscisic acid (ABA) during post-germinative stage. (A) Root elongation of wild type and transgenic plants exposed to ABA after germination. Three-day-old seedlings grown on 0.5X MS were transferred to 0.5X MS containing 0, 10, or 30 μM ABA. After 9 days, the representative images were taken. (B) Growth of wild-type and transgenic plants exposed to ABA after germination. Two-day-old seedlings grown on 0.5X MS were transferred to 0.5X MS containing 0 or 5 μM ABA. After 4 days, the representative images were taken, and the cotyledon greening in each line was measured. Data represent the mean ± standard error values obtained after evaluating 50 plants from three independent experiments. Different letters indicate significant differences (ANOVA; P < 0.05). [file Image_5.PDF]
