# Supplementary material for: Functional Analysis of the Pepper Ethylene-Responsive Transcription Factor, CaAIEF1, in Enhanced ABA Sensitivity and Drought Tolerance
Source: Front Plant Sci. 2017 Aug 22;8:1407. doi: 10.3389/fpls.2017.01407 (PMC5572256; doi:10.3389/fpls.2017.01407)
Supplement: FIGURE S2 — Comparisons of the deduced amino acid sequence of the CaAIEF1 protein with Capsicum annuum proteins. Identical amino acid residues are highlighted in black, and the upper line indicates the VIGS target region. [file Image_2.PDF]

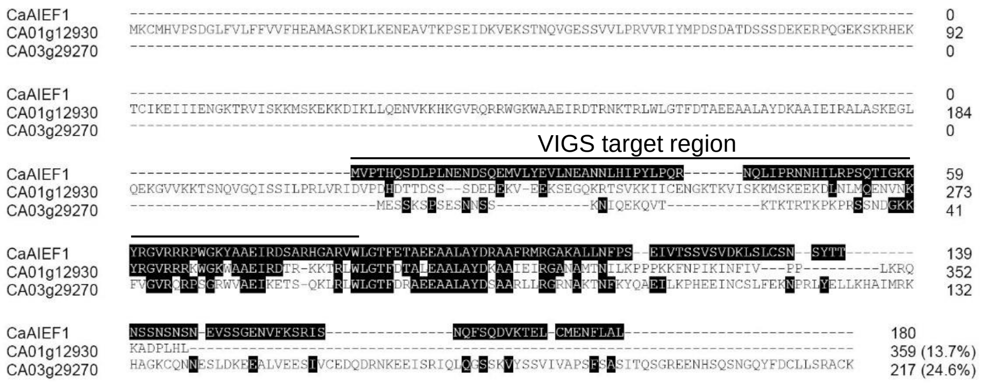

Supplementary Fig. S2. Hong et al.
