# Supplementary material for: Functional Analysis of the Pepper Ethylene-Responsive Transcription Factor, CaAIEF1, in Enhanced ABA Sensitivity and Drought Tolerance
Source: Front Plant Sci. 2017 Aug 22;8:1407. doi: 10.3389/fpls.2017.01407 (PMC5572256; doi:10.3389/fpls.2017.01407)
Supplement: Supplementary file 6 [file Table_1.PDF]

Table S1. Sequences of primers used in this study

Primer name	Primer sequence (5'-3')
For cloning	
CaDTEF1	Forward: ATGGTTCCAACTCACCAAAGTGAT
	Reverse: TCAGAGCGCCAAGAAATTCTC
For RT-PCR	
CaDTEF1	Forward: TGGTTCCAACTCACCAAAGTG
	Reverse: AACGCCTCTGTATTTCTTGCC
CaACT1	Forward: GACGTGACCTAACTGATAACCTGAT
	Reverse: CTCTCAGCACCAATGGTAATAACTT
CaNCED3	Forward: AGATTAGTTCAAGAACGTGAATTGG
	Reverse: ACTTGATAAGGGACATCATCTTCAG
CaLOX1	Forward: ACGTAATCTCTGGGAAAAATGACGAT
	Reverse: ACGTGACATCAAAGGCTGATTCGC
CaMLO2	Forward: ATGGCTAAAGAACGGTCGATGGA
	Reverse: GGTCATCATACTCAGACTTGACATCTTCAT
Actin8	Forward: CAACTATGTTCTCAGGTATTGCAGA
	Reverse: GTCATGGAAACGATGTCTCTTTAGT
NCED3	Forward: ACATGGAAATCGGAGTTACAGATAG
	Reverse: AGAAACAACAAACAAGAAACAGAGC
RD29A	Forward: CACAATCACTTGGCTCCACTGTTG
	Reverse: ACCTAGTAGCTGGTATGGAGGAACT
RD29B	Forward: GTTGAAGAGTCTCCACAATCACTTG
	Reverse: ATTAACCCAATCTCTTTTTCACACA
COR15A	Forward: GATACATTGGGTAAAGAAGCTGAGA
	Reverse: ACATGAAGAGAGAGGATATGGATCA
RAB18	Forward: GGAAGAAGGGAATAACACAAAAGAT
	Reverse: GCGTTACAAACCCTCATTATTTTTA
KIN1	Forward: TGTTAACTTCGTGAAGGACAAGAC
	Reverse: AAGTTTGGCTCGTCTAATAATTTTG
